# Unravelling the ORFan Puzzle

**DOI:** 10.1002/cfg.311

**Published:** 2003-07

**Authors:** Naomi Siew, Daniel Fischer

**Affiliations:** 1 Department of Chemistry Ben Gurion University Beer-Sheva 84105 Israel; 2 Bioinformatics Group Department of Computer Science Ben Gurion University Beer-Sheva 84105 Israel

## Abstract

ORFans are open reading frames (ORFs) with no detectable sequence similarity to any other sequence in the databases. Each newly sequenced genome contains a significant number of ORFans. Therefore, ORFans entail interesting evolutionary puzzles. However, little can be learned about them using bioinformatics tools, and their study seems to have been underemphasized. Here we present some of the questions that the existence of so many ORFans have raised and review some of the studies aimed at understanding ORFans, their functions and their origins. These works have demonstrated that ORFans are an untapped source of research, requiring further computational and experimental studies.


**Comparative and Functional Genomics**
*Comp Funct Genom* 2003; **4**: 432–441.
Published online in Wiley InterScience (www.interscience.wiley.com). **DOI:** 10.1002/cfg.311

**Conference Review**

### Unravelling the ORFan puzzle

Naomi Siew[1,2] and Daniel Fischer[2]*

[1] *Department of Chemistry, Ben Gurion University, Beer-Sheva 84105, Israel*
[2] *Bioinformatics Group, Department of Computer Science, Ben Gurion University, Beer-Sheva 84105, Israel*

**Correspondence to: Daniel Fischer, Bioinformatics Group, Department of Computer Science, Ben Gurion University, Beer-Sheva 84105, Israel. E-mail: dfischer@cs.bgu.ac.il*

Received: 7 May 2003
Revised: 5 June 2003
Accepted: 5 June 2003

### Abstract

**ORFans are open reading frames (ORFs) with no detectable sequence similarity to any other sequence in the databases. Each newly sequenced genome contains a significant number of ORFans. Therefore, ORFans entail interesting evolutionary puzzles. However, little can be learned about them using bioinformatics tools, and their study seems to have been underemphasized. Here we present some of the questions that the existence of so many ORFans have raised and review some of the studies aimed at understanding ORFans, their functions and their origins. These works have demonstrated that ORFans are an untapped source of research, requiring further computational and experimental studies.** **Copyright © 2003 John Wiley & Sons, Ltd.**

**Keywords:** **ORFans; evolution; functional genomics; power-law; structural genomics**

The availability of dozens of whole-genome sequences has given us new perspectives on our understanding of Nature's diversity and evolution, but it has also demonstrated that the interpretation of this information is a challenging problem. One interesting observation is the varying levels of conservation of open reading frames (ORFs) among the various genomes, mainly reflecting that the genetic material is mostly the result of the basic evolutionary process of descent with modification. Sequence families that are conserved among all (or most) known genomes correspond to proteins essential for life. Other families contain ORFs from organisms belonging to one kingdom only, and thus correspond to functions specific to that kingdom. In addition to these relatively conserved families, the currently fully sequenced genomes also contain a variety of families with decreasing levels of conservation. At the lower end, we observe a non-negligible number of families which contain lineage-specific ORFs (present in only a few, generally closely related organisms), or species- or strain-specific ORFs [47].

With the publication of almost every new genome, researchers have noted that a significant percentage (25–30%) of the ORFs in each new genome does not match any other ORF in the sequence databases [13,18,20,55]. Such ORFs have been referred to as orphans, singletons or ORFans for short [18]. The presence of so many ORFans suggests that sequence diversity in Nature may be greater than previously expected [10,20]. However, because little can be learned about ORFans via homology, each ORFan represents a mystery awaiting interpretation [9,14,16,18].

As our sequence databases are rapidly filling with more and more ORFans, studies aimed at elucidating the origin and functions of ORFans are sorely required. Although several groups are aiming at investigating the ORFan phenomenon, it seems that ORFans have received little attention overall [19]. Here we present some of the questions that the existence of so many ORFans has raised, and review the works that have demonstrated that ORFans are an untapped source of research, requiring further computational and experimental studies.

### Are ORFans a time-dependent artifact?

In order to study how the number of ORFans changes as new genomes are sequenced and added

Copyright © 2003 John Wiley & Sons, Ltd.

to the databases, we have carried out a dynamics analysis of ORFans among the first 60 fully sequenced microbial genomes [47,48]. Our data suggest that the large number of observed ORFans is not likely to be an artifact of sparse sampling. We have shown that every new sequenced genome has two effects on the total number of ORFans. First, the addition of each new genome slightly reduces the total number of older ORFans, because some of the latter find matches with ORFs of the new genome. The second effect is that each new genome adds a number of new ORFans. Because the number of new ORFans is usually larger than the number of older ORFans that become non-ORFans, the total number of ORFans in the database is constantly growing (Figure 1 in ref 47). Among the 60 genomes, containing a total of 168 248 ORFs, 23 634 (14%) are ORFan sequences (see also our ORFan database at **http://www.cs.bgu.ac.il/~nomsiew/ORFans**). Our dynamics analysis suggests that the number of ORFans is not likely to significantly drop in the near future. Indeed, almost every subsequently sequenced genome continues to include a large percentage of ORFans (with an astonishing 60% in *P. falciparum* [21]).

However, because older ORFans slowly find homologues in organisms that have been sequenced more recently, eventually, when a number of strains have been sequenced for each organism, many of today's ORFans may trivially become non-ORFans. To better identify those poorly conserved sequences and those genus- or species-specific sequences, broader definitions of ORFans are required.

### Broader ORFan definitions

A large number of conversions of older ORFans to non-ORFans occur when the genome of an organism which is closely related to a previously sequenced one is added to our database [47]. Thus, these older ORFans begin forming small families of homologues that match ORFs from closely related genomes only. To better address such groups of

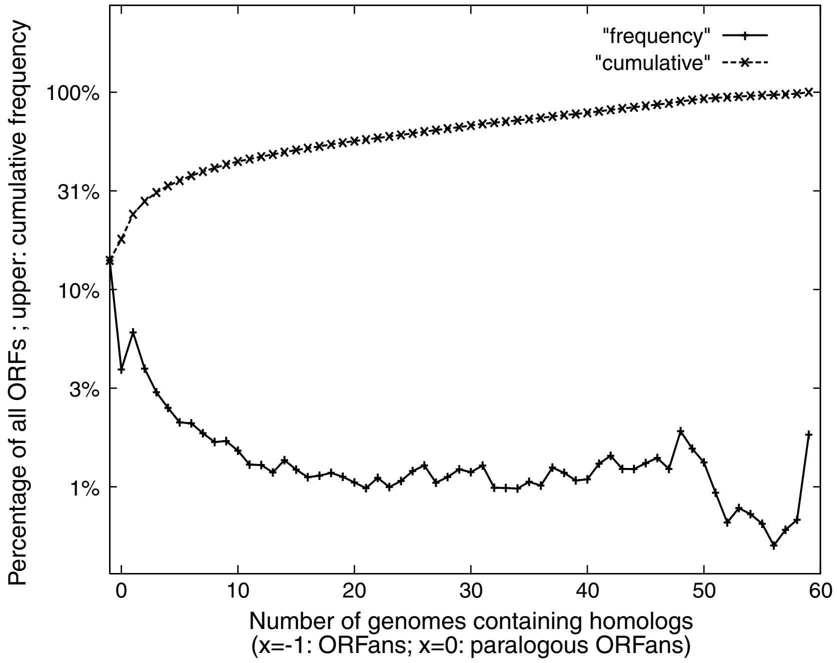

**Figure 1.** About one-third of all ORFs are poorly conserved. Frequency distribution of the homologies found among the 168 248 ORFs in our database of 60 genomes. For each ORF, we computed the number of genomes containing sequences homologous to it. The *x*-axis corresponds to the number of genomes. $x = -1$ corresponds to singleton ORFans (23 634); $x = 0$ corresponds to paralogous ORFans (6618). The *y*-axis shows the frequency in logarithmic scale. The upper line is the cumulative frequency. The peaks at $x = 48$ and $x = 59$ correspond to those ORFs that are conserved among most of the 50 bacteria in our database, and those conserved among all 60 genomes, respectively

Copyright © 2003 John Wiley & Sons, Ltd. 

proteins we have introduced the term 'orthologous ORFans' [47], which we define as those ORFs that have homologues only among closely related organisms and none outside.

Another term we have introduced is 'paralogous ORFans' [47], which refers to those ORFs that have homologues in the same organism (and thus, by definition, they are not ORFans) but none in other genomes. Notice that our notions of paralogy and orthology are different from the usual ones; we use them here merely to refer to those ORFs having homologues within one or a set of closely related organisms only, without any implications with regards to their evolutionary histories. To distinguish between paralogous and orthologous ORFans to those ORFans having no homologues whatsoever, we use the term 'singleton' ORFans [47]. A more accomodating term, which includes singleton, paralogous and orthologous ORFans, and which does not need a definition of what 'closely related organisms' are, is that of 'poorly-conserved ORFs', or PCOs (Shaanan, Eichler and Fischer, unpublished). We say an ORF is a PCO if all its homologues correspond to ORFs from a very small number of organisms. An interesting property of PCOs is their time- and sampling- independence. A PCO today, lacking homologues in the majority of currently known genomes, is likely to remain a PCO in the future.

Figure 1 shows the frequency distribution of ORFs in the first 60 microbial genomes as a function of the number of organisms in which each ORF finds homologues (i.e. for each ORF $o$, we count the number of organisms that contain sequences homologous to $o$). PCOs correspond to the first few values from the left: the first two correspond to singleton ORFans (at $x = -1$), and to paralogous ORFans (at $x = 0$), respectively. Orthologous ORFans appear at $x$ values $>1$. About one-third of all ORFs correspond to sequences having homologues in at most five genomes. Thus, PCOs correspond to a significant percentage of the genetic material.

### Do ORFans correspond to real genes?

A number of publications have claimed that a portion of ORFs may correspond to errors or misannotated genes (e.g. [16,18,34,36,37,46,49,54]). Especially dubious are the shorter ORFs (with less than 100–150 codons), which have been referred to as smORFs (for 'small ORFs') [8] or ELFs (for 'evil little fellows') [41]. SmORFs are problematic because, without evidence from homology to other ORFs, they may correspond to spurious, non-coding ORFs [4,8,38,41,49,54].

On the other hand, it has also been claimed that the majority of putative ORFs, even those that are short, are genuine protein-coding regions [33,41]. Furthermore, it is likely that due to misannotation, many important, functional smORFans have not even been identified [4,8]. We and others have claimed that only a minority of (the shorter) ORFans may not correspond to real genes [16,46,48]. Dujon and colleagues have shown that a majority of the ca. 3000 initial *Saccharomyces cerevisiae* ORFans (which they refer to as 'maverick genes') are actively transcribed [37]. They also show that a large number of the *S. cerevisiae* genes are Ascomycetes-specific ('orthologous ORFans' in our terminology), half of which have been functionally characterized. Experimental studies on individual ORFans from *Escherichia coli* [1] and *Halobacterium* NRC-1 (Shaanan, Eichler and Fischer, unpublished) have suggested that they correspond to real, expressed proteins. Large-scale transposon mutagenesis experiments on *Mycoplasma* [26] ORFs, including many ORFans, have not only suggested that a majority of them correspond to real proteins, but also that many correspond to essential proteins (see below). Evidence suggesting that many ORFans correspond to real proteins has also been obtained from other genomes not in our microbial genome database. For example, of the 1437 ORFans identified in the *Anopheles gambiae* genome (about 11% of all the ORFs) [57], over one-third are supported by expressed sequence tag matches. Of these 1437 ORFans, 522 correspond to paralogous ORFans. In addition to these, 579 orthologous ORFans have been identified within *Anopheles* and *Drosophila melanogaster*, which may help determine insect-specific features [57].

Further evidence that most ORFans correspond to functional proteins is obtained from our dynamics studies described above. The fact that older ORFans slowly find homologues in organisms that have been sequenced more recently, strongly suggests (but is not absolute proof [41]) that they do correspond to real, functional proteins [47]. For example, 11% of *Mycoplasma genitalium*'s ORFs originally corresponded to singleton

Copyright © 2003 John Wiley & Sons, Ltd.

ORFans, all of which became orthologous ORFans when *Mycoplasma pneumoniae* was added to the database. This suggests that these ORFans were present in the common *Mycoplasma* ancestor, and that throughout evolution they have been conserved in both genomes. After 60 genomes, no ORF from genomes not belonging to the *Mycoplasma* family matches any of these ORFans, suggesting that they correspond to proteins specific to the mycoplasmas.

Our analysis shows that both short and long ORFans can become non-ORFans. However, our results also suggest that some of the shorter ORFans may indeed correspond to non-proteins. Two observations of our dynamics analysis provide evidence for this. First, we have found that the rate at which longer ORFans become non-ORFans is about two-fold higher than that of the shorter ORFans. Second, we have found that about 60% of the current ORFans are short, and conversely, about 40% of the short ORFs are ORFans. This proportion is much higher than that observed for longer ORFs (only about 7% of the longer ORFs are ORFans) [47]. These findings suggest that some, but not all, of the shorter ORFans may indeed not correspond to genes.

Finally, one could claim that ORFans simply correspond to pseudogenes [3,38].We claim that very few (if any) of the longer ORFans may correspond to pseudogenes. Most pseudogenes are often identified through homology to functional genes (except for those that have degenerated beyond recognition) and thus they are not ORFans by definition. In our ORFan computations we only include ORFs that are not identified as pseudogenes. Furthermore, microbial genomes contain very few pseudogenes [3,35,38] (exceptions are e.g. *Rickettsia prowazekii* and *Mycobacterium leprae*), because bacteria maintain relatively high deletion rates (e.g. [39]). This deletion mechanism removes (alien and) non-functional material and prevents bacterial genomes from being filled with pseudogenes and other DNA [35]. The majority of the microbial genomes in our database are probably free from long pseudogenes and are also most likely free from other long, non-functional DNA segments that may have incorrectly been annotated as ORFs [35,43]. Thus, it is unlikely that most (longer) ORFans will correspond to pseudogenes or non-functional proteins. In summary, it seems that, with the exception of the shorter ones, most ORFans correspond to real genes.

### On the origin and functions of ORFans

The most common gene formation mechanism is the duplication of existing genes. The duplicated sequences can then diverge and subfunctionalization can occur, making both of the copies essential. Alternatively, one of the copies may gain a new function or fold into a new three-dimensional (3D) structure or may degenerate to a non-functional gene [53]. Regardless of the fate of the duplicates, their sequences may remain relatively similar (e.g. homologous), or they may diverge beyond recognition with current tools.

If ORFans are the result of this process, then one needs to explain why is it that they are so divergent from all other proteins, and why we do not see today any of the intermediate sequences that must have given rise to them [18,47]. Possible explanations may be rapid evolution [37,46,57] or massive gene-loss (e.g. [3,5,33,43,57]). However, because current tools are not able to detect the relationship of ORFans to other proteins, accepting their origin as being distant relatives of other proteins does not help us in characterizing and understanding them. Furthermore, this explanation about the origin of ORFans brings a number of other unexplained questions worth studying [45,47]. What are the mechanisms that control their rapid evolution? What are the evolutionary mechanisms that allow different evolutionary rates for different ORFs? What impact such processes have on the functions and structures of ORFans? Are there deletion mechanisms that operate in parallel? Have these highly divergent sequences lost their function, or have they acquired new functions and/or 3D-structures? If ORFans do correspond to functional proteins, why don't we see (near) duplicates? Are they non-essential or non-functional proteins on the way to extinction? If so, then it is clear that some mechanisms must exist that are responsible for their routine creation and deletion. Alternatively, if ORFans correspond to essential proteins, are they on the way to forming multi-membered families (see below)?

Another possible origin of ORFans may be lateral gene transfer between species (e.g. [15,29]),

Copyright © 2003 John Wiley & Sons, Ltd. 

possibly coupled with fast divergence. Nevertheless, knowing their possible origin does not help us in their characterization either; we do not yet know (or cannot identify) the sequence of the 'original' genomes that contributed these genes, and we may never know them. Furthermore, ORFans whose origin is the result of lateral transfer or gene loss [3,33] may be the only remnants of extinct families.

Finally, ORFans may be formed from existing DNA (e.g. [36,42]), or from non-coding DNA (*de novo* formation) [18,53]. This could be the origin of some of the short, non-coding ORFans (see above). Longer ORFans, if created *de novo*, are more likely to correspond to real genes. Although *de novo* gene formation is probably a very rare phenomenon (only a few examples have been reported), it is likely that every real gene created *de novo* will be an ORFan.

It remains to be seen which of the above (or other) mechanisms give rise to ORFans [47]. In any case, none of the above explanations about the origin of ORFans enables us to characterize them. Even if many ORFans turn out to be 'simply' distant members of known families, they may be interesting subjects of study. This is so because they will provide excellent examples of the subtleties of how highly divergent sequences retain, lose or acquire functionality [45,47]. In short, having possible explanations of the origin of ORFans only makes them more interesting to study, and still leaves us with an enormous amount of ORFans in the databases awaiting characterization.

### Are ORFans essential proteins?

To the best of our knowledge, only sporadic experimental analysis on a few ORFs have been carried out to address this question ([26,37,46] and see [38] for a recent review).

One may initially think that many ORFans must correspond to non-essential proteins because of the notion that essential proteins are more conserved in bacteria [30], and consequently the least conserved ones, and especially ORFans, may be non-essential. However, other studies (e.g. [24,25]) have not found any significant difference between the evolution rates of essential and non-essential genes. Large-scale studies in yeast have also demonstrated that the fraction of ORFans ('maverick' genes) with essential functions is not different from that of non-ORFans [37]. Global transposon mutagenesis has suggested that up to 60% of the orthologous *Mycoplasma* ORFans may correspond to essential proteins [26].

Being non-essential does not mean 'uninteresting' or non-functional. Non-essential proteins may be the drivers for the evolutionary diversification [31]. Many ORFans may turn out to correspond to the species determinants [18,28,31,38]. Further large-scale experimental characterization will not only determine the functionality and essentiality of ORFans, but they will also reveal whether ORFans correspond to new, unique proteins with novel functions or 3D structures not observed before. Thus, ORFans are particularly attractive targets for characterization.

### Prioritizing ORFan studies

Because experimental characterization is expensive and time-consuming, many studies focus first on the many sequence families containing homologues from numerous organisms. For example, large-scale structural genomics initiatives aim at structurally characterizing first those proteins that exist in a large number of organisms [11,51]. One goal of these projects is, through a careful selection of 10 000–20 000 targets, to provide a good coverage of structural space, so that we will be able to computationally model most of the remaining proteins using the solved targets (i.e. most of the other proteins will lie at the so-called 'homology-modelling' distance [17]). However, such estimates have not taken into account the large number of ORFans and PCOs whose structures we won't be able to computationally model using any of the solved targets (by definition, each ORFan lies beyond 'homology-modelling' distance from any other protein). Thus, to achieve a more complete coverage, in addition to the selected 10 000–20 000 targets, experimental structures will be needed for each ORFan and for at least one representative of each PCO family [48].

Because ORFans (and PCOs in general) account for such a large percentage of the genomic material, it is not too soon to begin their characterization. In

Copyright © 2003 John Wiley & Sons, Ltd.

addition, because ORFans may correspond to proteins with novel functions or 3D structures, they are attractive targets for crystallization [11,12,19]. Although few groups have initiated experimental projects dedicated to characterizing ORFans and PCOs ([1,6,22,40] and Shaanan, Eichler and Fischer, unpublished results from the *Halobacterium* NRC-1 ORFan project), major international efforts on ORFans and PCOs will be required before a more complete coverage of the structure space is achieved.

### Surprisingly many ORFans and PCOs?

A number of studies have shown that proteomes, like many real-life networks and complex systems, have properties similar to the so-called 'scale-free' networks [7,27,44,56]. One of the interesting properties of such networks is that they contain a small number of highly-connected nodes and a very large number of nodes with few connections [the distribution of the number of connections of the nodes follows a power (Zipf) law; also known as a 'power-law' distribution].

The interest in studying biological processes using mathematical tools such as the scale-free model stems from the fact that they can potentially provide a higher level of abstraction and can help observe features of biological importance that may not be easily detectable otherwise [52]. In our case, scale-free networks can help us generalize some of the properties of ORFans and PCOs and of the evolutionary relationships among ORFs. In particular, these studies demonstrate that the number of observed ORFans and PCOs is consistent with what could be expected from a scale-free network.

Very recently, Unger and colleagues [50] have extended previous works by showing that the distribution of sizes of protein families from a number of databases also follow a power-law behaviour governed by two exponents, one for the most connected families (superfamilies) and another for the least connected ones (PCOs). They also propose a simple model of protein evolution where proteins are dynamically generated and clustered into families, yielding similar distributions to those found in the real data, for the large and small families. They conclude that such a model suggests that the existence of superfamilies and ORFans are manifestations of the same evolutionary process. Similarly, Karev and colleagues [32] have proposed a model for the composition of domains in individual proteomes, which consists of domain birth, death and innovation. They show that the domain family size distributions on a number of proteomes are similar to those obtained from a particular, balanced form of their model.

We have performed similar analyses using the connectivity of ORFs from our database of 60 complete genomes at different points in time. Our analysis differs from previous works in that we analyse the distribution of the number of direct 'neighbours' that each ORF has in all genomes in our database, and not the distribution of family sizes within each genome. This may have some disadvantages, but has the advantage of removing the possible biases introduced by pre-computed clustering procedures.

Figure 2 shows the frequency distribution of the number of homologues each ORF has, computed after 8, 15, 22, 30, 45 and 60 genomes were added to our database of fully sequenced genomes. Notice that Figure 2 differs from Figure 1 in that here we count the number of neighbours (homologous sequences) each ORF has, regardless of the number of genomes in which these neighbours reside. Thus, an ORF with 10 neighbours may correspond to a paralogous ORFan having 10 neighbours in the same genome, or to an ORF having homologues in 10 different genomes.

The frequency of ORFans and PCOs (plotted at the very left) follows the trend of the ORFs with few neighbours (Figure 1). Thus, one may suggest that the number of observed ORFans is just what could be expected from this network. Figure 2, like Figure 1, shows that a large number of ORFs (58 344 ORFs, or 35% of all ORFs after 60 genomes) are poorly conserved, having less than a handful of homologues. A number of other interesting properties of these plots are described in the legend of Figure 2. It will be interesting to see whether the distributions observed here will hold after hundreds of genome sequences are available, and whether the proportion of ORFs with very few neighbours will remain as high. It also remains to be seen whether the resulting distributions will continue to show two or three breakpoints, or will acquire non-scale-free shapes.

Copyright © 2003 John Wiley & Sons, Ltd.

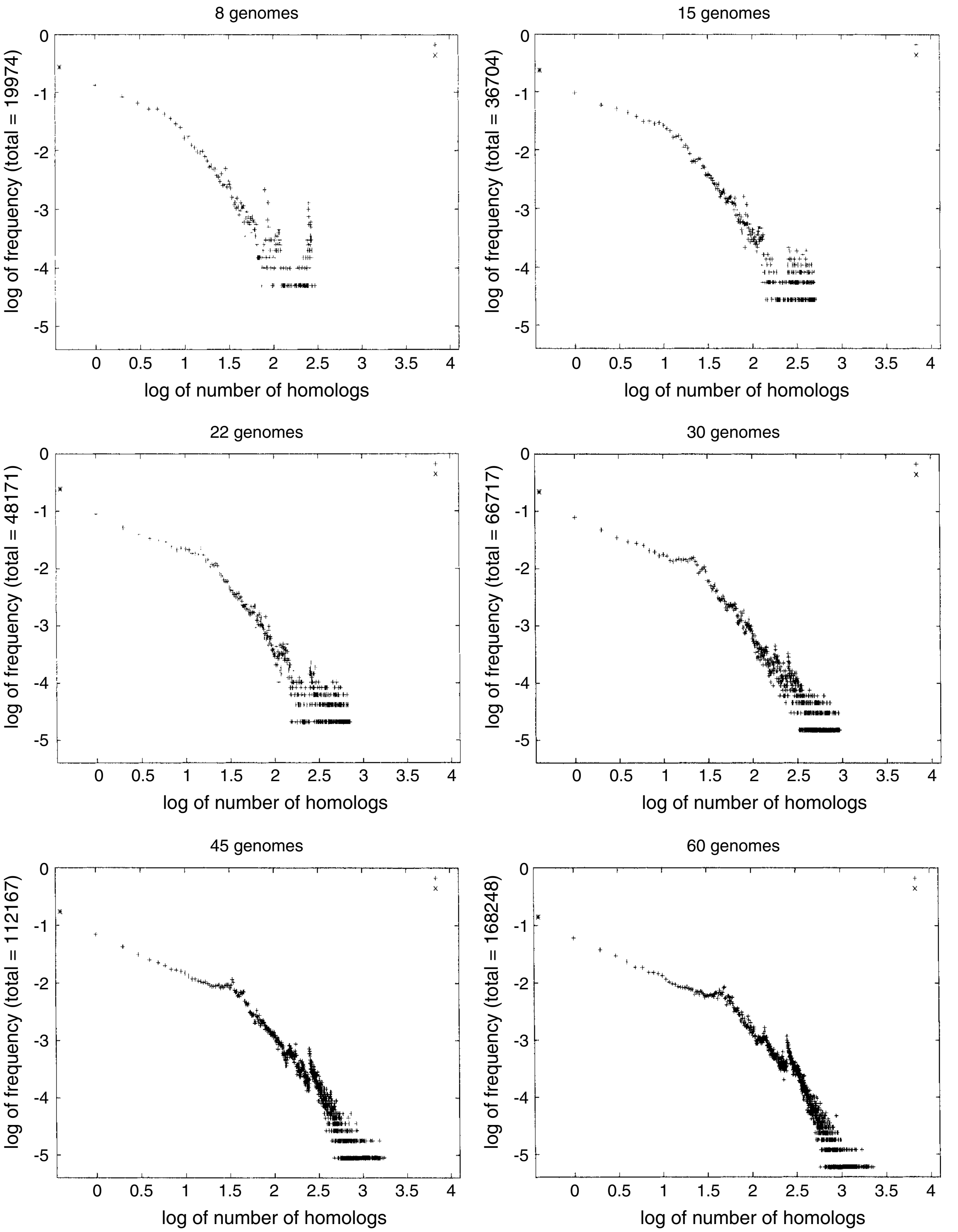

8 genomes log of frequency (total = 19974)

log of number of homologs

15 genomes log of frequency (total = 36704)

log of number of homologs

22 genomes log of frequency (total = 48171)

log of number of homologs

30 genomes log of frequency (total = 66717)

log of number of homologs

45 genomes log of frequency (total = 112167)

log of number of homologs

60 genomes log of frequency (total = 168248)

log of number of homologs

### Discussion

We propose the following model to explain the origin and abundance of ORFans and PCOs, which is somewhat consistent to the models discussed above. Many ORFans may have been generated as the result of a number of possible evolutionary events, which may include horizontal transfer, rapid evolution and gene-loss. ORFans (and other ORFs) without selection pressure have been deleted throughout microbial deletion mechanisms, and thus, microbial genomes are kept at 'reasonable sizes' [43]. ORFans that have retained or acquired an important function are kept, thus creating new sequence families with a seed of a single ORFan. With time, and subsequent duplications, this family may expand to form a family of paralogues, or may remain as a singleton family, if no advantage is gained by the generation of near-duplicates (as is the case for a number of proteins, e.g. ribosomal proteins). ORFans and PCOs observed today in the sequenced genomes may thus be the result of a dynamic process that may have occurred in the past, or may be ongoing; the genome sequence of a descendant may have some ORFans deleted, some new paralogous ORFans, and may contain a number of new ORFans (as has already been observed from the genome sequences of two strains of the same species, e.g. [2,23] and [38] for other examples). This suggests that even very closely related organisms can present significant diversity.

Not every functional ORFan will be the seed of a multi-membered paralogous family. Some ORFans may correspond to ancient proteins that do not proliferate (like the ribosomal proteins) and are likely to remain ORFans in the future. Our model does not imply that all genomes are currently experiencing (or have recently experienced) the above dynamic process. Some genomes may have reached some level of stability and their ORFans are likely to remain as functional, single-membered families. For example, because all the ORFs of *M. genitalium* show homology to ORFs from *M. pneumoniae*, it is clear that *Mycoplasma*'s orthologous ORFans originated before their divergence from their common ancestor and that *M. genitalium* has only

**Figure 2.** Log–log plots of the distribution of the number of neighbours (homologous sequences) of ORFs in our database at different points in time. The *x*-axis corresponds to the log of the number of neighbours each ORF has. The *y*-axis corresponds to the log of the frequency of ORFs at each value of *x* (i.e. the log of the number of ORFs with *x* neighbours, divided by the total number of ORFs in the database at each point in time). ORFans have no neighbours and are depicted in the plots as the left-most stars. Because of the log–log plot, ORFans can not be assigned an *x*-coordinate. We arbitrarily assigned them slightly to the left of $x = 0$. The plots at different times show that the percentage of ORFans is slowly declining. However, as noted above, the total number of ORFans is continuously increasing. It is clear that the shapes of the plots change significantly for the first 22 genomes, indicating that with fewer than 22 genomes, the sampling of sequence space may be too sparse. After 22 genomes a clear difference between the frequencies of the ORFs with few neighbours and those with many neighbours begin to appear. It is approximately at this point, when there were about 20 complete genomes, that the analysis of Unger and colleagues [50] was carried out. Consistent with their results, the distribution shows two tendencies, one for the ORFs with >20 neighbours (around 1.5 in the *x*-axis) and another for those with fewer than 20. The slope values for these two lines are very similar to those found by Unger (about −0.5 for the frequencies of the ORFs with few neighbours, and about −2.0 for those with many neighbours). Notice that the break-point (where the slope of the plot changes) occurs approximately at the *x*-value corresponding to the number of genomes considered. In the plots corresponding to >30 genomes, a second break point begins to appear (approximately at an *x*-value of 2.5, which corresponds to a degree of 500). It also seems that the slope for the less connected ORFs is flattening and that of the highly connected ORFs is increasing. Another interesting observation is that the average number of homologues per ORF is very close to the number of genomes considered. The distribution after 60 genomes clearly suggests three types of ORFs: ORFs with fewer than 60 neighbours ($x = 1.77$); ORFs having between 60 and 500 neighbours ($x = 2.5$); and ORFs having more than 500 neighbours. A possible explanation for these breakpoints is paralogy. Those ORFs with >500 neighbours (total, 1692, or 1% of all ORFs) correspond to proteins belonging to the most conserved families which have many paralogues in each genome. Not surprisingly, the 20 ORFs with the largest number of neighbours (1745–2238 homologues) correspond to ABC transporters, which are present as large families in all genomes. Almost 80% of these 1692 ORFs have neighbours in all 60 genomes. The other peak (at about 60 neighbours) corresponds to highly conserved ORFs that appear as single copies in each genome. Examples of these are ribosomal proteins and tRNA synthetases. ORFs with <60 neighbours (total, 121 460) correspond to the vast majority of all ORFs (72%). 80% of these 121 460 ORFs have neighbours in less than 24 genomes and 58 344 ORFs (35% of all ORFs) have neighbours in at most five genomes. The remaining 44 308 ORFs (having 60–500 neighbours) correspond to a mixture of highly conserved proteins belonging to families with various degrees of paralogy, and to proteins of large paralogous families that are not present in all of the genomes (e.g. present only in bacteria)

Copyright © 2003 John Wiley & Sons, Ltd. 

experienced deletions since then. All these orthologous ORFans without paralogues are likely to correspond to relatively old proteins which do not produce advantageous paralogues in either genome. *Rickettsia prowazekii* and *Mycobacterium leprae*, with a significant number of pseudogenes [3,35], are exceptional examples suggesting that their deletion mechanisms no longer succeed in maintaining 'clean' genomes.

The delicate balance of the rates of generation and deletion is responsible for the maintenance of a compact, clean genome and at the same time, allows the organism to efficiently explore the vast sequence space to generate diversity. The abundance of ORFans and PCOs is merely a consequence of this balance. Any change in the rate of generation/deletion may compromise survival. ORFans are simply the result of a natural evolutionary process and their number is exactly what would be expected from a scale-free system. Thus, in addition to the classical view of 'duplication with modification', the proposed model may be responsible for the enormous microbial diversity.

Further computational and experimental ORFan studies (with emphasis on the longer paralogous and orthologous ORFans), will allow us to verify the validity of this model and will also provide answers to the questions of the origin of ORFans, of how many of them correspond to real genes, to essential proteins or to proteins with novel functions or novel 3D structures.

### Acknowledgements

This joint research project was partially supported by Grant No. 81948101 from the United States-Israel Binational Science Foundation (BSF), Jerusalem, Israel. N.S. is supported by a grant from the Ministry of Science, Israel, and by the Kreitman Foundation Fellowship.

### References

1. Alimi JP, Poirot O, Lopez F, Claverie J-M. 2000. Reverse transcriptase-polymerase chain reaction validation of 25 'orphan' genes from *Escherichia coli* K-12 MG1655. *Genome Res* **10**(7): 959–966.
2. Alm RA, Ling LS, Moir DT, *et al.* 1999. Genomic-sequence comparison of two unrelated isolates of the human gastric pathogen *Helicobacter pylori*. *Nature* **397**: 176–180.
3. Andersson JO, Andersson SGE. 2001. Pseudogenes, junk DNA and the dynamics of *Rickettsia* genomes. *Mol Biol Evol* **18**: 829–839.
4. Andrade MA, Daruvar A, Casari G, *et al.* 1997. Characterization of new proteins found by analysis of short open reading frames from the full yeast genome. *Yeast* **13**(14): 1363–1374.
5. Aravind L, Watanabe H, Lipman DL, Koonin EV. 2000. Lineage-specific loss and divergence of functionally linked genes in eukaryotes. *Proc Natl Acad Sci USA* **97**: 11 319–11 324.
6. Balasubramanian S, Schneider T, Gerstein M, Regan L. 2000. Proteomics of *Mycoplasma genitalium*: identification and characterization of unannotated and atypical proteins in a small model genome. *Nucleic Acids Res* **28**: 3075–3082.
7. Barabasi AL, Albert R. 1999. Emergence of scaling in random networks. *Science* **286**: 509–512.
8. Basrai MA, Hieter P, Boeke JD. 1997. Small open reading frames: beautiful needles in the haystack. *Genome Res* **7**: 768–771.
9. Bloom BR. 2000. On the particularity of pathogens. *Nature* **406**: 760.
10. Boucher Y, Nesbo CL, Doolittle WF. 2001. Microbial genomes: dealing with diversity. *Curr Opin Microbiol* **4**: 285–289.
11. Brenner SE. 2000. Target selection for structural genomics. *Nature Struct Biol* **7**(suppl): 967–969.
12. Coulson AFW, Moult J. 2002. A unifold, mesofold, and superfold model of protein fold use. *Proteins* **46**: 61–71.
13. Doolittle RF. 1997. A bug with excess gastric avidity. *Nature* **388**: 515–516.
14. Doolittle RF. 2002. Microbial genomes multiply. *Nature* **416**: 697–700.
15. Doolittle WF. 1999. Phylogenetic classification and the universal tree. *Science* **284**: 2124–2129.
16. Dujon B. 1996. The Yeast Genome Project: what did we learn? *Trends Genet* **12**(7): 263–270.
17. Fischer D, Baker D, Moult J. 2001. We need both computer models and experiments [correspondence]. *Nature* **409**: 558.
18. Fischer D, Eisenberg D. 1999. Finding families for genomic ORFans. *Bioinformatics* **15**: 759–762.
19. Fischer D. 1999. Structural genomics: affirmative action for ORFans and the growth in our structural knowledge. *Prot Eng* **12**: 101–102.
20. Fraser CM, Eisen JA, Salzberg SL. 2000. Microbial genome sequencing. *Nature* **406**: 799–803.
21. Gardner MJ, Shallom SJ, Cartton JM, *et al.* 2002. Genome sequence of the human malaria parasite *Plasmodium falciparum*. *Nature* **419**: 498–511.
22. Goulding CW, Parseghian A, Sawaya MR, *et al.* 2002. Crystal structure of a major secreted protein of *Mycobacterium tuberculosis MPT63* at 1.5Å resolution. *Protein Sci* **11**(12): 2887–2893.
23. Hayashi T, Makino K, Ohnishi M, *et al.* 2001. Complete genome sequence of enterohemorrhagic *Escherichia coli* O157:H7 and genomic comparison with a laboratory strain K-12. *DNA Res* **8**(1): 11–22.
24. Hirsh AE, Fraser HB. 2001. Protein dispensability and rate of evolution. *Nature* **411**: 1046–1049.
25. Hurst LD, Smith NG. 1999. Do essential genes evolve slowly? *Curr Biol* **9**: 747–750.
26. Hutchison CA III, Peterson SN, Gill SR, *et al.* 1999. Global transposon mutagenesis and a minimal *Mycoplasma* genome. *Science* **286**: 2165–2169.

Copyright © 2003 John Wiley & Sons, Ltd. 

27. Huynen MA, van Nimwegen E. 1998. The frequency distribution of gene family sizes in complete genomes. *Mol Biol Evol* **15**: 583–589.
28. Iliopoulos I, Tsoka S, Andrade MA, *et al.* 2000. Genome sequences and great expectations. *Genome Biol* **2**(1): interactions 0001.1–0001.3.
29. Jain R, Rivera MC, Lake JA. 1999. Horizontal gene transfer among genomes: the complexity hypothesis. *Proc Natl Acad Sci USA* **96**: 3801–3806.
30. Jordan IK, Rogozin IB, Wolf YI, Koonin EV. 2002. Essential genes are more evolutionarily conserved than are non-essential genes in bacteria. *Genome Res* **12**(6): 962–968.
31. Jordan IK, Rogozin IB, Wolf YI, Koonin EV. 2002. Microevolutionary genomics of bacteria. *Theor Popul Biol* **61**(4): 435–447.
32. Karev GP, Wolf YI, Rzhetsky AY, Berezovskaya FS, Koonin EV. 2002. Birth and death of protein domains: a simple model of evolution explains power law behavior. *BMC Evol Biol* **2**: 18.
33. Koonin EV. 2001. Computational genomics. *Curr Biol* **10**: R155–R158.
34. Kunin V, Cases I, Enright AJ, de Lorenzo V, Ouzounis CA. 2003. Myriads of protein families, and still counting. *Genome Biol* **4**: 401.
35. Lawrence JG, Hendrix RW, Casjens S. 2001. Where are the pseudogenes in bacterial genomes? *Trends Microbiol* **9**: 535–540.
36. Mackiewicz P, Kowalczuk M, Gierlik RM, Dudek A, Cebrat S. 1999. Origin and properties of non-coding orfs in the yeast genome. *Nucleic Acids Res* **27**: 3503–3509.
37. Malpertuy A, Tekaia F, Casaregola S, *et al.* 2000. Genomic exploration of the hemiascomycetous yeasts: 19. Ascomycetes-specific genes. *FEBS Lett* **487**: 113–121.
38. Mira A, Klasson L, Andersson SGE. 2002. Microbial genome evolution: sources of variability. *Curr Opin Microbiol* **5**: 506–512.
39. Mira A, Ochman H, Moran NA. 2001. Deletional bias and the evolution of bacterial genomes. *Trends Genet* **17**: 589–596.
40. Monchois V, Abergel C, Sturgis J, Jeudy S, Claverie J-M. 2001. *Escherichia coli ykfE* ORFan gene encodes a potent inhibitor of C-type lysozyme. *J Biol Chem* **276**: 18 437–18 441.
41. Ochman H. 1996. Distinguishing the ORFs from the ELFs: short bacterial genes and the annotation of genomes. *Trends Genet* **18**: 325–327.
42. Pellegrini M, Yeates TO. 1999. Searching for frameshift evolutionary relationships between protein sequence families. *Proteins* **37**: 278–283.
43. Petrov DA, Sangster TA, Johnston JS, Hartl DL, Shaw KL. 2000. Evidence for DNA loss as a determinant of genome size. *Science* **287**: 1060–1062.
44. Qian J, Luscombe NM, Gerstein M. 2001. Protein family and fold occurrence in genomes: power-law behaviour and evolutionary model. *J Mol Biol* **313**: 673–681.
45. Rost B. 2002. Did evolution leap to create the protein universe? *Curr Opin Struct Biol* **12**: 409–416.
46. Schmid KJ, Aquadro CF. 2001. The evolutionary analysis of ‘orphans’ from the *Drosophila g*enome identifies rapidly diverging and incorrectly annotated genes. *Genetics* **159**: 589–598.
47. Siew N, Fischer D. 2003. Analysis of singleton ORFans in fully sequenced microbial genomes. *Proteins* (in press).
48. Siew N, Fischer D. 2003. Twenty thousand ORFan microbial protein families for the biologist? *Structure* **11**(1): 7–9.
49. Skovgaard M, Jensen LJ, Brunak S, Ussery A, Krogh D. 2001. On the total number of genes and their length distribution in complete microbial genomes. *Trends Genet* **17**: 425–428.
50. Unger R, Uliel S, Havlin S. 2003. Scaling-law in sizes of protein sequence families: from super-families to orphan genes. *Proteins* **51**: 569–576.
51. Vitkup D, Melamud E, Moult J, Sander C. 2001. Completeness in structural genomics. *Nature Struct Biol* **8**(6): 559–566.
52. Wolf YI, Karev G, Koonin EV. 2002. Scale-free networks in biology: new insights into the fundamentals of evolution? *BioEssays* **24**: 105–109.
53. Wolfe KH, Li WH. 2003. Molecular evolution meets the genomics revolution. *Nature Genet* **33**: (suppl): 255–265.
54. Wood V, Rutherford KM, Ivens A, Rajandream M-A, Barrell B. 2001. A re-annotation of the *Saccharomyces cerevisiae* genome. *Comp Funct Genom* **2**: 143–154.
55. Wren BW. 2000. Microbial genome analysis: insights into virulence, host adaptation and evolution. *Nature Rev Genet* **1**: 30–39.
56. Yanai I, Camacho CJ, DeLisi C. 2000. Predictions of gene family distributions in microbial genomes: evolution by gene duplication and modification. *Phys Rev Lett* **85**: 2641–2644.
57. Zdobnov EM, von Mering C, Letunic I, *et al.* 2002. Comparative genome and proteome analysis of *Anopheles gambiae* and *Drosophila melanogaster*. *Science* **298**: 149–159.

Copyright © 2003 John Wiley & Sons, Ltd. 
